# From kratom to 7-hydroxymitragynine: evolution of a natural remedy into a public-health threat

**DOI:** 10.1080/13880209.2025.2590311

**Published:** 2025-11-23

**Authors:** Scott Alsbrook, George Pro, Igor Koturbash

**Affiliations:** ^a^ Department of Environmental Health Sciences, Fay W. Boozman College of Public Health, University of Arkansas for Medical Sciences, Little Rock, AR, USA; ^b^ Department of Health Behavior and Health Education, Fay W. Boozman College of Public Health, University of Arkansas for Medical Sciences, Little Rock, AR, USA; ^c^ Center for Dietary Supplements Research, Fay W. Boozman College of Public Health, University of Arkansas for Medical Sciences, Little Rock, AR, USA

**Keywords:** 7-OH, 7-hydroxymitragynine, kratom, mytragynine, opioids

## Abstract

**Context:**

Kratom *(Mitragyna speciosa),* native to Southeast Asia, has traditionally been consumed as fresh leaves or teas. Under those conditions, exposure to 7-hydroxymitragynine (7-OH)—a potent μ-opioid receptor agonist—is minimal, as it occurs only at trace levels in leaf material. By contrast, the U.S. market offers chemically enriched or semi-synthetic 7-OH products, often marketed as ‘kratom’ yet chemically distinct from botanical preparations.

**Methods:**

‘7-OH’, ‘7-hydroxymitragynine’, and ‘kratom’ were used as keywords; relevant literature was obtained from PubMed, Web of Science, and Google Scholar.

**Results:**

Pharmacological studies consistently identify 7-OH as a partial μ-opioid receptor agonist with nanomolar affinity, greater efficacy than mitragynine, and often exceeding the potency of morphine. Animal experiments demonstrate robust antinociceptive effects, respiratory depression, tolerance, dependence, and reinforcing properties characteristic of opioids. Human pharmacokinetic studies show systemic exposure after kratom ingestion, but concentrated 7-OH products bypass metabolic formation, producing markedly higher exposures. Regulatory surveillance, poison-center data, and marketplace audits confirm a rapid increase in availability and use of these products. State health departments have reported severe intoxications and fatalities. Clinical cases describe escalating use, medically managed withdrawal, and psychiatric destabilization, while forensic investigations document postmortem concentrations consistent with fatal opioid toxicity. Pediatric risk is amplified by developmental susceptibility, absence of age restrictions, and marketing in confectionary formats. Emerging analogues such as MGM-15 further extend this trajectory.

**Conclusion:**

Collectively, the evidence demonstrates that concentrated 7-OH products are pharmacologically and toxicologically distinct from kratom leaf and pose significant risks of morbidity and mortality under typical conditions of use.

## Introduction

Kratom (*Mitragyna speciosa* Korth., Rubiaceae) is a tropical evergreen tree indigenous to Southeast Asia—southern Thailand, Malaysia, Indonesia, and surrounding regions (Hossain et al. 2023; Heywood et al. 2024; Begum et al. 2025). Botanically related to coffee, kratom thrives in wetland areas and has been traditionally cultivated for its psychoactive and medicinal properties (Heywood et al. 2024; Begum et al. 2025). The earliest Western descriptions of kratom use date back to the early nineteenth century, but ethnobotanical evidence indicates that local communities have used it for centuries. In its traditional context, fresh leaves were chewed to stave off fatigue among laborers or brewed into teas to relieve pain, diarrhea, and fever (Hossain et al. 2023; Heywood et al. 2024). The traditional mode of administration was largely confined to water decoctions or chewing fresh leaves, which limited alkaloid intake compared with contemporary preparations (Heywood et al. 2024).

Kratom began appearing in U.S. markets in the late twentieth century, with state public health reports noting introduction by the late 1990s and broader commercial expansion occurring after the mid-2000s (Henningfield et al., 2024; McCurdy et al. 2024; Virginia Department of Health, 2024). In the U.S., kratom is marketed primarily through online vendors, convenience stores, and specialty shops. Its products now include powders, capsules, tablets, concentrated extracts, gummies, and energy drinks (Heywood et al. 2024; Grundmann et al. 2025). These formulations contrast sharply with traditional Southeast Asian use and enable higher, more concentrated, and more frequent dosing (Heywood et al. 2024; Begum et al. 2025; Vadiei et al. 2025). Contemporary marketing emphasizes self-treatment for chronic pain, anxiety, depression, fatigue, and opioid withdrawal (Heywood et al. 2024; McCurdy et al. 2024). National survey data suggest that pain relief, relaxation, stress reduction, and energy enhancement are the leading reasons for use, with kratom often positioned as a ‘natural’ alternative to prescription opioids or stimulants (Green et al. 2025; Grundmann et al. 2025).

## Methods

Literature was identified through structured searches of PubMed, Web of Science Core Collection, and Google Scholar, supplemented by targeted web searches. Database queries combined controlled vocabulary and free-text terms for kratom and its alkaloids (e.g., ‘Mitragyna speciosa’, ‘7-hydroxymitragynine’, ‘7-OH’, ‘mitragynine pseudoindoxyl’), with field limits applied to title/abstract where available. Searches were last updated October 1, 2025, and yielded 2,216 unique records after automatic and manual de-duplication across databases. Titles and abstracts were screened for relevance to chemistry, pharmacology, toxicology, clinical cases, surveillance, or market characterization; full texts were obtained for records meeting inclusion criteria, which encompassed peer-reviewed articles and conference proceedings. Governmental and public-health materials (state department of health alerts/advisories, federal assessments) and contemporaneous media reports were identified *via* Google using structured strings (‘7-hydroxymitragynine’ OR ‘7-OH’ AND alert/advisory/health department/state) and restricted to the United States. Data were extracted into standardized templates capturing study type, analytic methods, key quantitative findings, and limitations; when multiple reports described the same event, the most detailed or primary source was prioritized. Non–peer-reviewed sources were used only to contextualize market practices or public-health actions and are cited as such. No language filters were imposed at the search stage, but only English-language sources were included in the analysis. Limitations include potential under-ascertainment of emerging products not yet indexed, heterogeneity of analytic panels that may miss 7-OH, and incomplete age-stratified data in public-health summaries.

## From mitragynine to 7-hydroxymitragynine: phytochemical and metabolic pathways

Phytochemically, kratom leaves contain a complex array of indole and oxindole alkaloids with more than 40 identified to date (Hossain et al. 2023). The major constituent is mitragynine, typically comprising 12–66% of the total alkaloid content, depending on geography, harvest, and processing conditions (Pohanka 2023). Mitragynine is metabolized to 7-hydroxymitragynine (7-OH), a minor natural alkaloid but pharmacologically far more potent at the μ-opioid receptor (Obeng et al. 2022; Chiang et al. 2025). Other significant alkaloids include speciogynine, speciociliatine, paynantheine, and corynantheidine, which display variable affinities for opioid, adrenergic, and serotonergic receptors (Green et al. 2025). Together, these alkaloids account for kratom’s biphasic effects: stimulant-like at low doses and sedative/analgesic at higher doses (Heywood et al. 2024).

Among kratom’s phytochemicals, 7-OH warrants particular attention. Contemporary analytical surveys of commercial and laboratory preparations indicate that 7-OH generally constitutes <2% of the total alkaloid content in kratom leaves and products (Kerrigan and Basiliere 2022; Heywood et al. 2024; Chiang et al. 2025). Expressed on a dry-weight basis rather than as a fraction of alkaloids, measured concentrations of 7-OH are low as well: for dried leaves and powders, reported ranges span approximately 0.011–0.039% *w/w* (0.114–0.393 mg/g), with many products below these upper bounds; liquid extracts typically contain ≤0.01 mg/g (Chiang et al. 2025). These quantitative observations are consistent with controlled human pharmacokinetic work and clinical reviews noting that 7-OH is not measurable in fresh leaves and is ordinarily encountered only at trace levels in traditional preparations relative to mitragynine (Huestis et al. 2024; McCurdy et al. 2024).

The scarcity of 7-OH in native plant matrices reflects both biosynthetic constraints and post-harvest chemistry. Multiple sources concur that fresh kratom leaves do not contain measurable 7-OH, whereas transformation from mitragynine to 7-OH can occur during post-harvest drying and processing, yielding low-level artifact formation that increases under oxidative conditions (Heywood et al. 2024; Chiang et al. 2025). *In vivo*, 7-OH is also formed as a phase-I oxidative metabolite of mitragynine via intestinal and hepatic cytochrome P450 enzymes, a pathway demonstrated in both preclinical and human studies (Obeng et al. 2022; Chiang et al. 2025). Thus, 7-OH in consumer products may originate from three routes: innate, low-abundance plant occurrence; *ex vivo* conversion of mitragynine during drying or processing; and endogenous biotransformation after ingestion.

Beyond these natural routes, 7-OH can be obtained by semi-synthetic conversion of mitragynine in the laboratory. Experimental pharmacology reports routinely employ 7-OH prepared by chemical oxidation of mitragynine, and such preparations are used as reference ligands in receptor-binding and behavioral studies (Matsumoto et al. 2014). Recent marketplace analyses and regulatory assessments further document the emergence of commercial non-prescription products in which 7-OH is produced by chemical conversion of mitragynine isolates or extracts, yielding formulations with greatly enriched 7-OH content relative to natural leaf (Hill et al. 2025; Smith et al. 2025; Vadiei et al. 2025). These products, often labeled or marketed alongside ‘kratom’ despite distinct composition, appear as sublingual tablets, gummies, sirups, nasal sprays, and shots, and in some cases are advertised to circumvent first-pass metabolism, thereby increasing systemic exposure compared with traditional decoctions or ground-leaf capsules (US Food and Drug Administration 2025). In contrast, traditional Southeast Asian use centered on chewing fresh leaves or consuming aqueous decoctions, practices that inherently limited delivery of 7-OH given its minimal presence in fresh material and the modest yields from water extraction (Heywood et al. 2024; Huestis et al. 2024).

### 7-Hydroxymitragynine on the U.S. market

The emergence of concentrated 7-OH products in the United States is recent and distinguishable from the earlier commercialization of kratom leaf and extracts. Scholarly analyses of online retail activity indicate that explicitly labeled 7-OH and 7-OH/mitragynine-pseudoindoxyl formulations appeared on vendor sites in approximately 2024 and expanded through early 2025, after which hundreds of distinct items were cataloged across gummies, sublingual or chewable tablets, liquid ‘shots’, sirups, nasal sprays, and other formats (Hill et al. 2025; Smith et al. 2025; Vadiei et al. 2025). This pattern contrasts with the two prior decades during which U.S. kratom commerce was dominated by dried leaf powders, capsules, and hydroalcoholic extracts (Heywood et al. 2024; McCurdy et al. 2024). Converging evidence from regulatory surveillance and independent product audits describes a ‘proliferation’ of high-potency 7-OH offerings during this interval, supported by poison center and law-enforcement toxicology signals consistent with increased human exposure to concentrated 7-OH products (Smith et al. 2025; US Food and Drug Administration 2025). Although formal time-series prevalence estimates for 7-OH specifically are not yet available, cross-sectional market characterizations and federal assessments concur on an upward trajectory in availability relative to the historical absence of such products before 2024 (Hill et al. 2025; Vadiei et al. 2025).

Distribution channels initially centered on online specialty vendors but rapidly expanded into convenience retail. Clinical case documentation describes ready access to 7-OH tablets at gas stations and smoke shops, paralleling the distribution of kratom shots and extracts and highlighting the transition from niche online sales to over-the-counter availability (Smith et al. 2025). Federal review similarly notes widespread internet and brick-and-mortar sales of concentrated 7-OH products despite the absence of any approved drug indications and despite the compound’s potent μ-opioid receptor pharmacology (US Food and Drug Administration 2025).

Marketing claims for 7-OH products encompass general effects such as ‘focus’ and ‘relaxation’ and extend to functional assertions implying therapeutic use, including ‘pain relief’ and ‘anxiety reduction’, often presented without distinction from botanical kratom (Hill et al. 2025; Vadiei et al. 2025). A systematic web audit found that most 7-OH items made effect claims, and more than one-third made functional health claims; many were couched in language typical of kratom promotion, and product names sometimes alluded to prescription opioids (Hill et al. 2025). Independent commentaries caution that such positioning may mislead consumers unfamiliar with the chemical and pharmacological differences between 7-OH and kratom leaf, particularly given that 7-OH is a potent μ-opioid agonist and that some formulations are designed for sublingual or intranasal use, thereby bypassing first-pass metabolism and amplifying systemic exposure (Vadiei et al. 2025; US Food and Drug Administration 2025). The U.S. Food and Drug Administration has emphasized that 7-OH is a trace botanical constituent typically absent in fresh leaves and that most commercial preparations are generated through chemical oxidation of mitragynine isolates, yielding enriched formulations that do not resemble natural kratom decoctions or ground-leaf products (US Food and Drug Administration 2025).

The terminological conflation of semi-synthetic 7-OH products with ‘kratom’ is a consistent theme across independent sources. Marketplace analyses observed that a substantive fraction of 7-OH and 7-OH/mitragynine-pseudoindoxyl offerings were labeled or marketed under the ‘kratom’ umbrella, despite compositions (up to nearly pure 7-OH in some cases) that diverge markedly from native leaf alkaloid profiles (Hill et al. 2025; Smith et al. 2025). Regulatory assessments likewise identify a pattern in which concentrated 7-OH products are advertised alongside kratom and imply that consumers may incorrectly infer safety and historical-use equivalence with botanical preparations (US Food and Drug Administration 2025; Vadiei et al. 2025). This exploitation of kratom nomenclature is consequential because national survey data indicate that U.S. kratom users most commonly report self-managing pain, stress, and energy with leaf-based powders, pills, and teas (Grundmann et al. 2025). Applying those expectations to high-potency 7-OH formulations risks mismatched dosing and opioid-like adverse outcomes (Smith et al. 2025; Vadiei et al. 2025).

Taken together, these findings demonstrate a clear divergence between traditional kratom preparations and modern enriched 7-OH products. The growing presence of 7-OH on the U.S. market warrants better understanding of its pharmacology and toxicology.

## Basic pharmacology & metabolism

Across convergent *in vitro* evidence, 7-OH is consistently characterized as a potent μ-opioid receptor (MOR) agonist with substantially greater efficacy and affinity than mitragynine and, in several assays, greater functional potency than morphine. In rat and human membrane binding systems, 7-OH displays Ki values in the low-nanomolar to sub-hundred-nanomolar range at MOR, with robust agonist activity in [35S]GTPγS binding and tissue-based bioassays (Takayama et al. 2002; Matsumoto et al. 2014; Kerrigan and Basiliere, 2022; Obeng et al. 2022). By comparison, κ- and δ-opioid receptor interactions are much weaker, with evidence of neutral or antagonist activity depending on preparation (Kruegel et al. 2016). In electrically elicited contraction assays, such as guinea-pig ileum and mouse *vas deferens*, 7-OH suppresses neurotransmission via opioid-sensitive mechanisms, with effects reversed by naltrexone—hallmarks of MOR mediation (Matsumoto et al. 2014).

Side-by-side comparisons with mitragynine emphasize this pharmacological divergence. Mitragynine exhibits low-to-moderate MOR activity and engages adrenergic-α2 and serotonergic targets, producing a mixed receptor profile, whereas 7-OH acts as a selective MOR agonist across assays (Kerrigan and Basiliere 2022; Obeng et al. 2022). Isobolographic and antagonist-defined studies further confirm that 7-OH’s antinociceptive effects are abolished by naltrexone but not by α2-adrenergic blockade, in contrast to mitragynine’s mixed mechanism (Takayama et al. 2002; Matsumoto et al. 2014). The U.S. Food and Drug Administration’s technical assessment aggregated binding data across the literature and concluded that 7-OH demonstrates consistent nanomolar MOR binding and functional full agonism (US Food and Drug Administration 2025). More recent analyses extend these findings: 7-OH displays ten- to twentyfold higher MOR binding affinity than mitragynine and comparable efficacy to classical opioids such as oxycodone, with *in vitro* signaling capacity approaching fentanyl in some assays (Chakraborty et al. 2021; Qu et al. 2023).

Pharmacokinetic (PK) studies complement these receptor-level findings. In dedicated experiments in Sprague–Dawley rats, 7-OH was rapidly absorbed, showing high permeability in Caco-2 cells monolayers and moderate plasma protein binding (∼73%) (Chiang et al. 2025). Hepatic extraction ratios ranged from ∼0.3 in microsomes to ∼0.6 in intact hepatocytes, consistent with intermediate first-pass metabolism. Intravenous dosing revealed clearance of ∼4.0 L/h/kg and a volume of distribution of ∼2.7 L/kg, values typical of a lipophilic MOR agonist with appreciable tissue distribution (Chiang et al. 2025). Oral dosing produced quantifiable plasma concentrations, confirming systemic bioavailability despite first-pass loss. These data are highly relevant to emerging consumer formulations, particularly in regard to sublingual and intranasal products, because circumvention of first-pass metabolism would be expected to increase early plasma exposure relative to traditional capsules or teas (US Food and Drug Administration 2025; Vadiei et al. 2025).

Human observations derive primarily from clinical pharmacology studies of encapsulated kratom leaf and controlled administration of mitragynine, with 7-OH detected as a metabolite. After oral kratom, plasma 7-OH appears as a secondary metabolite formed by intestinal and hepatic CYP-mediated oxidation of mitragynine, with CYP3A4 and CYP2D6 identified as principal enzymes (Obeng et al. 2022). Plasma levels are low relative to mitragynine, but due to greater MOR potency, 7-OH contributes materially to pharmacodynamic outcomes (Huestis et al. 2024). Controlled dosing studies of dried leaf document systemic exposure consistent with formation-rate limitation, whereby circulating 7-OH is constrained by mitragynine metabolism rather than direct ingestion (Huestis et al. 2024). Interindividual differences in CYP activity contribute to variability in plasma 7-OH concentrations, aligning with metabolic phenotypes seen for other opioids. Notably, recent work demonstrates that 7-OH can undergo a rapid, nonenzymatic rearrangement in human plasma to mitragynine pseudoindoxyl, a μ-opioid receptor agonist with greater potency and efficacy than its precursor. This finding has two implications: mitragynine pseudoindoxyl may contribute to *in vivo* pharmacodynamics after kratom or 7-OH intake, and *ex vivo* conversion during sample handling can confound quantitative interpretation if not controlled analytically (Kamble et al. 2020).

Collectively, receptor binding and PK data situate 7-OH as a compound with dual significance: under traditional use, it functions as a metabolite-driven contributor to kratom’s pharmacology, but in the context of concentrated formulations it emerges as the primary driver of opioid-like effects. This distinction provides a mechanistic basis for subsequent discussions of toxicology and risk, as enriched 7-OH products shift the balance from peripheral contribution to central pharmacological dominance.

## Toxicology and animal studies

A critical point that emerges across the literature is that the toxicological profiles of traditional kratom preparations (mitragynine-dominant, with trace 7-OH formed *in vivo*) and concentrated 7-OH products are not equivalent. Reviews of botanical safety and hepatotoxicity situate kratom within a broader class of botanicals where reported adverse events often involve idiosyncratic liver injury, product heterogeneity, and poly-exposures (Ahmad et al. 2021; Gurley et al. 2022; Koturbash et al. 2024). In contrast, the 7-OH literature consistently shows a classical opioid-type signal—high antinociceptive potency, naltrexone-reversible effects, and hallmark opioid liabilities when exposure is sufficient—reflecting its role as a direct μ-opioid receptor (MOR) agonist delivered in concentrated form.

### In vivo studies

Multiple preclinical paradigms converge on robust opioid-like actions of 7-OH with high antinociceptive potency, MOR specificity, and classical liabilities of tolerance, physical dependence, and withdrawal. In rats, 7-OH produces dose-dependent antinociception in thermal nociception assays; its effects are antagonized by naltrexone and not by α2-adrenergic blockade, distinguishing it from mitragynine’s mixed mechanism (Matsumoto et al. 2014). In the same studies, 7-OH decreased operant responding for food in a naltrexone-reversible manner without α2-adrenergic involvement, again consistent with MOR agonism (Matsumoto et al. 2014). Hypothermia patterns further differentiate targets: reference α2-agonists produce yohimbine-reversible hypothermia, whereas 7-OH does not display α2-mediated thermoregulation; mitragynine, by contrast, shows α2-linked hypothermia (Matsumoto et al. 2014). These antagonist-defined pharmacological fingerprints reinforce 7-OH’s MOR selectivity *in vivo*.

### Comparative potency vs. classical opioids

Summaries in recent peer-reviewed reviews and the FDA’s technical assessment note that 7-OH shows multifold greater potency than morphine in antinociception paradigms, a pattern echoed in early and subsequent studies (Kerrigan and Basiliere 2022; Takayama et al. 2002; Matsumoto et al. 2014; US Food and Drug Administration 2025). Although fold-differences vary by assay, the consistent theme is superior functional potency of 7-OH at MOR relative to morphine and far greater than mitragynine (Obeng et al. 2022; US Food and Drug Administration 2025). Importantly, respiratory depressant action, a sentinel opioid toxicity, has been documented for 7-OH in animal studies at doses below equianalgesic morphine exposure, aligning it mechanistically with higher-risk opioids (US Food and Drug Administration 2025).

### Tolerance, dependence, withdrawal, and reward-related behaviors

With repeated administration, 7-OH exhibits the cardinal features of opioid tolerance and physical dependence in animals (Kerrigan and Basiliere 2022). FDA’s analysis of the preclinical literature notes development of tolerance to antinociceptive effects, escalating dose requirements, and the emergence of withdrawal signs either spontaneously upon cessation or precipitated by naloxone/naltrexone—patterns mirroring morphine, fentanyl, oxycodone, and hydrocodone (US Food and Drug Administration 2025). Reward-related behaviors, including conditioned place preference and self-administration, have been demonstrated for kratom alkaloids with emphasis on 7-OH’s MOR agonism and on mitragynine’s metabolic conversion to 7-OH *in vivo*; intracranial self-stimulation and allied paradigms show opioid-like reductions in brain-stimulation reward thresholds consistent with abuse liability when 7-OH exposure is sufficient (Obeng et al. 2022; US Food and Drug Administration 2025).

### Kratom vs. 7-OH: aligning toxicology with exposure context

The broader kratom context provides mechanistic plausibility for differing risk profiles depending on route and composition. Traditional aqueous decoctions and chewed fresh leaves contain little or no 7-OH at baseline; thus, opioid-type liabilities are attenuated and depend primarily on metabolic formation from mitragynine (Obeng et al. 2022; Heywood et al. 2024; Huestis et al. 2024). In contrast, semi-synthetic 7-OH products deliver high amounts of the MOR agonist directly, at times *via* sublingual or intranasal routes that increase early systemic availability and bypass first-pass metabolism (US Food and Drug Administration 2025). The alignment of animal data with these formulation differences supports the concern that concentrated 7-OH preparations will carry higher opioid-type risks than botanical kratom (US Food and Drug Administration 2025; Vadiei et al. 2025).

While the focuses here is given to an opioid-class toxicities, it is important to locate 7-OH within the broader toxicological landscape of kratom. Contemporary reviews highlight that hepatotoxicity signals associated with kratom in the literature are heterogeneous, often idiosyncratic, and frequently confounded by multi-ingredient products or co-exposures, aligning kratom with other herbal dietary supplements that show sporadic drug-induced liver injury (DILI) or cardiac toxicity rather than predictable dose-dependent hepatic toxicity (Schimmel and Dart 2020; Ahmad et al. 2021; Gurley et al. 2022; Koturbash et al. 2024; Alameh et al. 2025; Miller et al. 2025). This pattern stands in contrast to 7-OH’s predictable, dose-related opioid pharmacodynamics, which bring respiratory depression, tolerance, dependence, and withdrawal to the foreground when 7-OH is delivered in concentrated amounts. In short: kratom’s principal non-opioid toxicology concern has often centered on DILI or cardiac toxicity under specific, sometimes confounded circumstances, whereas 7-OH’s principal toxicology concern is opioid-class toxicity, with risk magnitude tightly coupled to formulation and dose.

### Emerging nuances (metabolism, variability, and sex)

Metabolic formation of 7-OH from mitragynine (CYP3A4, CYP2D6; intestinal and hepatic) introduces inter-individual variability that can modulate opioid-type effects even with botanical products (Basiliere and Kerrigan 2020; Kamble et al. 2020; Obeng et al. 2022; Huestis et al. 2024). In animal work with direct 7-OH dosing, PK differences (absorption, clearance, protein binding ∼73%, distribution volume ∼2.7 L/kg) and bioavailability (quantifiable after oral administration) help explain why formulations that reduce first-pass loss (sublingual/intranasal) show more pronounced early effects (Chiang et al. 2025; US Food and Drug Administration 2025). Preliminary rodent evidence also suggests sex-dependent differences in sensitivity to 7-OH’s behavioral and physiological effects, a nuance that warrants systematic study as the market continues to evolve.

Collating the animal and mechanistic data leads to a coherent picture: under traditional kratom use, opioid-type risks are moderated by low baseline 7-OH and reliance on metabolic formation; under concentrated 7-OH exposure, risks shift toward a classical opioid profile (naltrexone-reversible antinociception, respiratory depression, tolerance, dependence, withdrawal), with hazard magnitude amplified by dose and route.

## Surveillance and clinical evidence of emerging 7-hydroxymitragynine risk

Population-level data specific to 7-OH remain limited; however, triangulation from regulatory surveillance, marketplace audits, poison center reporting, and clinical observations indicates a sharp recent increase in availability and exposure in the United States. The U.S. Food and Drug Administration’s 2025 technical assessment documented a proliferation of concentrated 7-OH products newly marketed as chewable or sublingual tablets, shots, gummies, sirups, and nasal sprays, many produced by chemical oxidation of mitragynine and sold through both online and brick-and-mortar outlets (US Food and Drug Administration 2025). A structured web audit conducted between late 2024 and early 2025 identified 304 distinct 7-OH and mitragynine-pseudoindoxyl products, with more than 80% being 7-OH–only and most labeled for sublingual or transmucosal use; effect claims such as ‘focus’ and ‘relaxation’ were ubiquitous, and more than one-third of products carried functional health claims (Hill et al. 2025).

These marketplace findings are corroborated by FDA’s aggregation of multiple surveillance streams, including America’s Poison Centers’ National Poison Data System, law-enforcement toxicology programs, and social-media monitoring, all showing increased mentions and exposures consistent with rising real-world use of concentrated 7-OH (US Food and Drug Administration 2025). Importantly, earlier surveillance rarely assayed for 7-OH specifically, relying instead on mitragynine as a surrogate marker for ‘kratom’. This approach risks misclassification when exposures derive from semi-synthetic 7-OH products containing only trace mitragynine (Smith et al. 2025). FDA emphasizes that this analytic gap likely led to under-recognition of 7-OH involvement in prior poisoning reports and fatalities, complicating trend analysis (US Food and Drug Administration 2025; Smith et al. 2025). Only recently have laboratories and surveillance networks begun to implement targeted testing for 7-OH and related semi-synthetic alkaloids, enabling differentiation from botanical kratom (US Food and Drug Administration 2025).

State-level surveillance reinforces the national picture. In Texas, the Department of State Health Services reported that, as of August 27, 2025, the Texas Poison Center Network had documented 192 exposures involving kratom or products containing 7-OH—already surpassing totals for all of 2024 (107) and 2023 (122). Nineteen 2025 cases specifically involved concentrated 7-OH, and eleven required medical treatment (Texas Department of State Health Services 2025). In Pennsylvania, the Health Alert Network summarized kratom and 7-OH cases reported between January 1, 2022, and June 30, 2025: patients ranged in age from 12 months to 80 years; 49 were female and 117 male. Abuse accounted for the largest share (71 cases), followed by misuse (22), withdrawal (12), and suspected suicide (10). Clinical severity was notable: 81 individuals experienced significant illness, naloxone was administered in 25 cases, and 14 required mechanical ventilation, although no deaths were reported (Pennsylvania Department of Health 2025).

Local alerts have highlighted fatal outcomes. On September 12, 2025, the Los Angeles County Department of Public Health issued an alert describing multiple fatal overdoses linked to concentrated 7-OH, emphasizing that such products, usually marketed as tablets, gummies, and liquid extracts, were readily available in local convenience outlets and online. At least three fatalities were explicitly attributed to 7-OH, often in conjunction with alcohol use, with naloxone responsiveness observed but sometimes requiring repeated dosing (Los Angeles County Department of Public Health 2025).

Epidemiologic interpretation remains complicated by the conflation of concentrated 7-OH products with ‘kratom’ in both retail labeling and consumer discourse. The National Survey on Drug Use and Health reported 1.6 million adults used kratom in 2023 (Center for Behavioral Health Statistics and Quality 2025). National survey data on kratom use document pills, gummies, and powders used primarily for pain relief, relaxation, stress reduction, and energy enhancement (Grundmann et al. 2025). Yet these surveys do not distinguish botanical kratom from semi-synthetic 7-OH, leaving uncertainty as to how much of the reported use pertains to concentrated formulations. This misclassification risk is amplified by marketing practices that frame 7-OH products as kratom, thereby importing consumer expectations from leaf-based powders and teas to high-potency MOR agonists (Hill et al. 2025; Smith et al. 2025; US Food and Drug Administration 2025).

Clinical case observations provide further depth to these epidemiologic signals. A detailed report described a 38-year-old man with prior opioid use disorder who transitioned from high-dose kratom capsules to over-the-counter 7-OH tablets purchased at gas stations and smoke shops; his use escalated to as many as eight 30-mg tablets every one to two hours (≈240 mg/day), with expenditures up to $60/day. On cessation he developed a syndrome of anxiety, insomnia, rhinorrhea, abdominal pain, restlessness, diaphoresis, and chills, consistent with opioid withdrawal, and presented for medically supervised detoxification (Wightman and Hu 2025). This case underscores the dependency-forming potential of concentrated 7-OH products and their accessibility outside of regulated pharmacy channels.

A contrasting presentation was described in a 56-year-old man who developed acute mania following abrupt kratom withdrawal. Symptoms included severe agitation, insomnia, aggression, grandiosity, and compulsive behavior; he required intensive care admission and psychiatric stabilization. The authors emphasized this as the first documented case of mania secondary to kratom withdrawal, expanding the clinical spectrum of kratom-related psychiatric morbidity beyond classic opioid toxidromes (Abidali et al. 2025).

Another report detailed a catastrophic neuropsychiatric outcome: a 31-year-old man with concurrent cannabinoid, mitragynine, and 7-OH exposure who developed substance-induced psychosis culminating in bilateral auricular and penile self-amputation (Broul et al. 2025). Though atypical of opioid toxicity, the case illustrates severe destabilization and self-harm linked to kratom alkaloids in the context of polysubstance use.

Most recently, Pullman et al. (2025) reported a 29-year-old man with prior opioid and alcohol use disorders who suffered cardiopulmonary arrest after ingesting approximately 190 mg of 7-OH in tablet form while intoxicated with alcohol. He required 10 min of cardiopulmonary resuscitation and was successfully revived with two intravenous doses of naloxone. The patient disclosed habitual 7-OH use at doses far exceeding labeled recommendations, and chemical analysis of the implicated product confirmed not only 7-OH but also mitragynine, mitragynine pseudoindoxyl, and other alkaloids, raising further concerns about undeclared composition. Despite polysubstance co-exposures, the prompt reversal with naloxone strongly suggested opioid-driven toxicity (Pullman et al. 2025).

Taken together, these cases delineate recurring features: non-pharmacy retail access to concentrated 7-OH formulations, presentations consistent with MOR agonism and opioid withdrawal, but also psychiatric destabilization, catastrophic self-harm, and in at least one case, near-fatal cardiopulmonary arrest. They highlight not only the opioid-like risks of 7-OH but also the potential for psychiatric complications and underscore the role of polysubstance exposure (alcohol, cannabinoids, stimulants) in amplifying morbidity. Overall, the emerging epidemiologic picture is one of rapid expansion in concentrated 7-OH availability and exposure. Multiple surveillance streams—marketplace audits, poison center data, state and county alerts, and published case reports—converge on the conclusion that 7-OH products have shifted kratom from a primarily botanical commodity into the domain of a potent opioid-like public health hazard.

## Forensic & postmortem human data

Forensic and postmortem investigations provide direct toxicological evidence linking 7-OH to morbidity and mortality, though interpretation is often complicated by polysubstance exposures and the historical absence of targeted 7-OH testing. Early reports anticipated the emerging risk profile. A 2014 Norwegian case described a middle-aged man found deceased with postmortem blood concentrations of mitragynine 1.06 mg/L and 7-OH 0.15 mg/L, in the presence of therapeutic-range zopiclone, citalopram, and lamotrigine (Karinen et al. 2014). The authors concluded that mitragynine and 7-OH likely contributed to death, noting pulmonary findings consistent with opioid overdose and recommending routine inclusion of both alkaloids in forensic panels for suspected kratom fatalities.

Subsequent large-scale studies have reinforced these concerns. Osawa and Johnson (2025) analyzed 51 autopsy cases in which mitragynine and 7-OH were both quantified. In most cases 7-OH was present at lower levels than mitragynine, consistent with its role as a metabolite. However, in a subset of cases—particularly those associated with concentrated 7-OH products—postmortem 7-OH concentrations were disproportionately high, suggesting direct ingestion of enriched formulations rather than metabolic formation. The study emphasized the challenge of relying on mitragynine as a surrogate marker for ‘kratom’ in toxicology reports, as such practice risks under-recognizing fatalities driven primarily by 7-OH.

Local forensic alerts underscore these findings. In September 2025, the Los Angeles County Department of Public Health reported three confirmed fatalities in otherwise healthy young adults, all attributed to concentrated 7-OH products obtained in retail outlets and online (see case discussed above) (Los Angeles County Department of Public Health 2025). The advisory warned that product labeling often obscured 7-OH content, with items sold under general descriptors such as ‘plant alkaloids’ or ‘kratom alkaloids’.

Additional clinical–forensic overlap is illustrated by a case described by Pullman et al. (2025), in which a 29-year-old man suffered cardiopulmonary arrest after ingesting a concentrated 7-OH product in the context of alcohol co-use. He was resuscitated with cardiopulmonary support and naloxone, and laboratory analysis of the implicated tablets confirmed 7-OH together with other kratom alkaloids, including mitragynine and mitragynine-pseudoindoxyl. Although the patient survived, the combination of product testing, clinical course, and analytical confirmation situates this case firmly within the forensic toxicology domain and underscores the lethality potential of concentrated 7-OH formulations.

The American College of Medical Toxicology’s ToxIC Novel Opioid and Stimulant Exposure (NOSE) report (2025) provided a sentinel case of a 45-year-old woman who initially survived ingestion of a white powder labeled ‘kratom’ but later died after returning in cardiac arrest. Toxicology confirmed mitragynine, 7-OH, caffeine, amphetamine, and yohimbine. The clinical presentation at first contact was judged consistent with opioid toxidrome, and the report concluded that 7-OH may have been a principal driver despite polysubstance involvement (ACMT ToxIC NOSE 2025).

Taken together, forensic and postmortem data corroborate the signals observed in epidemiology and clinical case reports: 7-OH can produce fatal opioid toxicity, often in contexts where consumers assume they are using kratom products of botanical origin. These cases consistently highlight three interrelated challenges. First, polysubstance use (most commonly alcohol, stimulants, or sedatives) complicates causal inference but does not preclude a central role for 7-OH when the clinical course and quantitative findings support an opioid toxidrome. Second, historical reliance on mitragynine-only testing has created a substantial attribution gap, leading to underestimation of 7-OH involvement in both nonfatal and fatal events. Third, 7-OH is unstable in human plasma and can undergo a rapid, nonenzymatic rearrangement to mitragynine pseudoindoxyl, a μ-opioid receptor agonist with greater potency and efficacy than its precursor; without attention to preanalytical conditions, *ex vivo* conversion can bias quantitation and obscure the true distribution of active opioid species (Kamble et al. 2020).

These considerations argue for targeted toxicology that reflects the chemical and metabolic realities of kratom alkaloids. Routine panels in clinical and postmortem casework should include mitragynine, 7-OH, and mitragynine pseudoindoxyl, together with commonly reported phase I/II metabolites characterized in human matrices, to distinguish botanical kratom exposure from concentrated or semi-synthetic 7-OH products, and to detect artifactual rearrangement when handling conditions are imperfect (Philipp et al. 2009; Kamble et al. 2020). Where short-window matrices are unavailable or confounded, keratinized matrices (hair, nails) can document medium-term exposure and complement case attribution and surveillance (Ameline et al. 2023; Rhee et al. 2024). Implementing these analytic updates will reduce misclassification, improve comparability across laboratories, and better resolve the contribution of 7-OH to morbidity and mortality in contemporary case series.

Overall, forensic evidence aligns with regulatory surveillance in underscoring that concentrated 7-OH is not simply a stronger version of kratom but a pharmacologically distinct entity whose toxicological risks mirror those of classical opioids.

### 7-Hydroxymitragynine and pediatric populations

The pediatric population represents a particularly vulnerable group in the context of kratom and, more specifically, 7-OH exposure. Several factors converge to create heightened risk, including developmental neurobiology, the absence of regulatory safeguards in many jurisdictions, and deliberate marketing practices that increase youth appeal.

From a pharmacological standpoint, children and adolescents exhibit greater susceptibility to opioid agonists due to immature hepatic and renal metabolic systems, differences in blood–brain barrier permeability, and ongoing brain development in reward and executive function circuits. These features increase the probability of exaggerated responses to potent μ-opioid receptor agonists such as 7-OH and raise the risk of rapid reinforcement, tolerance, and subsequent dependence relative to adults (US Food and Drug Administration 2025; Obeng et al. 2022). Preclinical evidence that 7-OH exceeds morphine in analgesic potency and produces robust opioid-like dependence syndromes underscores the potential severity of pediatric exposure (Matsumoto et al. 2014; US Food and Drug Administration 2025).

Compounding these intrinsic vulnerabilities are deficiencies in regulatory oversight. In many U.S. states, kratom and kratom-containing products, including those adulterated or enriched with 7-OH, can legally be sold without age restrictions (Heywood et al. 2024; US Food and Drug Administration 2025). Adolescents are thus able to purchase such products at gas stations, smoke shops, and convenience stores. Parallel availability through online commerce further facilitates unsupervised acquisition by teenagers. Marketplace analyses confirm extensive internet sales of 7-OH products with effect claims including relaxation, focus, and pain relief, frequently marketed under the broader ‘kratom’ label (Hill et al. 2025; Smith et al. 2025). For minors with unrestricted internet access, these platforms provide ready entry points for experimentation.

Marketing strategies exacerbate pediatric risk. A consistent observation across product audits and regulatory assessments is the use of brightly colored packaging and confectionary formats (gummies, chewables, candy-like tablets), which are inherently attractive to children and adolescents (Hill et al. 2025; Smith et al. 2025). A recent market analysis of mitragynine pseudoindoxyl and 7-OH products found that 69% featured child-appealing flavors or scents and 63% used bright colors in packaging or labeling (White et al. 2025). Such presentation obscures the pharmacological nature of the products and fosters misperceptions of safety, increasing the likelihood of accidental or intentional ingestion. Poison-control surveillance has already documented pediatric kratom exposures, although most historical monitoring did not specifically assay for 7-OH (US Food and Drug Administration 2025).

To date, the peer-reviewed literature does not include a pediatric case with analytical confirmation of 7-OH as the primary intoxicant. The indexed pediatric report describes a 15-year-old with kratom overdose and discusses kratom alkaloids (mitragynine and 7-OH) without definitive confirmation of 7-OH (Wong and Mun 2020). State-level advisories in 2025 report rising pediatric calls related to kratom and 7-OH, yet publicly available summaries do not specify 7-OH-only cases (America’s Poison Centers 2005; Pennsylvania Department of Health 2025; Texas Department of State Health Services 2025). Notably, the Pennsylvania Department of Health documented a kratom/7-OH exposure in a 12-month-old child (the youngest reported to date) underscoring that very young children are also at risk (Pennsylvania Department of Health 2025). The lack of differentiation between botanical kratom and semi-synthetic 7-OH products continues to complicate attribution, while the proliferation of concentrated 7-OH formulations suggests that pediatric cases will increasingly involve this high-potency alkaloid (Hill et al. 2025; Smith et al. 2025; US Food and Drug Administration 2025).

Analogous vulnerability has been documented with other non-approved psychoactives available online. For example, a focused review of phenibut intoxications described clusters of adolescent cases, including a series in which five teenagers required intubation, and noted that children constituted the largest subgroup of unintentional exposures reported to U.S. poison centers. The authors highlighted how youth-appealing presentation and easy e-commerce access disproportionately harm minors (Gurley and Koturbash 2024). In these regards, the parallels with 7-OH marketing and accessibility are direct and concerning.

Together, these observations indicate that although analytically confirmed pediatric cases of 7-OH intoxication remain undocumented, the pharmacological vulnerability of children, the absence of age-based regulation, and the youth-oriented marketing of concentrated 7-OH products converge to create substantial risk.

### Appearance of new molecules on the market—the MGMs

MGM-15 and MGM-16 are semi-synthetic indoloquinolizine opioids developed from 7-OH as part of medicinal chemistry programs to generate orally active dual μ- and δ-opioid receptor agonists. Both preserve the 7-OH indoloquinolizine core but differ in substitution: MGM-16 carries a 9-fluoro substituent, whereas MGM-15 is the corresponding non-fluorinated analogue (Matsumoto et al. 2014).

*In vitro*, MGM-15 and MGM-16 function as high-affinity, full μ/δ agonists in radioligand binding and [35S]GTPγS assays, suppressing electrically evoked contractions in guinea-pig ileum and mouse *vas deferens* in a naltrexone-reversible manner (Matsumoto et al. 2014). Comparative studies show greater potency for MGM-16, with Ki values at μ- and δ-receptors of ≈2.1 and 7.0 nM, respectively, while MGM-15 demonstrates somewhat weaker but still sub-hundred-nanomolar affinity. At recombinant human receptors, MGM-15 shows intermediate-nanomolar affinity for hMOR (≈28 nM) and hDOR (≈59 nM), exceeding 7-OH in the same assays (Gour et al. 2025).

*In vivo*, both molecules are orally active in mice and produce antagonist-defined opioid antinociception and antiallodynia. MGM-16 demonstrates striking potency, up to 71-fold greater than morphine by subcutaneous dosing and over 200-fold greater after oral administration in the tail-flick assay. MGM-15 is consistently less potent but still active and orally efficacious (Matsumoto et al. 2014). Blockade studies with β-funaltrexamine (μ) and naltrindole (δ) confirm that both act through dual-receptor mechanisms. While dedicated toxicology studies of MGM-15 are not yet available, its μ/δ agonism implies classical opioid liabilities. Broader reviews of δ-agonists note class-level concerns with side effects and limited clinical translation, raising similar questions for MGM-15 (Gour et al. 2025).

Relative to 7-OH, MGM-15 demonstrates higher μ/δ affinity and greater antinociceptive potency in rodents while retaining oral activity, features that initially motivated its exploration as a potential analgesic lead (Matsumoto et al. 2014; Gour et al. 2025). Although less potent than MGM-16, MGM-15 is synthetically simpler and more scalable, factors that may explain its emergence in consumer markets.

By 2025, MGM-15 had appeared in U.S. online vendor listings as ‘research-only’ or ‘reference’ material, usually sold as high-purity powder (≈97–99%) or less commonly as pre-metered tablets. Vendors typically displayed certificates of analysis, disclaimers (‘not for human consumption’), and extensive shipping exclusions reflecting varied state rules. Powder packages ranged from 250 mg to multi-gram lots, with unit-dose tablets around 12 mg. Finished products such as flavored liquids commanded higher prices. Site taxonomy sometimes placed MGM-15 under ‘7-hydroxymitragynine’ categories, reinforcing the terminological conflation between botanical kratom and semi-synthetic analogues (Gour et al. 2025). In contrast, MGM-16 has not been observed for online purchase, likely reflecting its exceptional potency, greater liability concerns, and added synthetic complexity (Matsumoto et al. 2014; Gour et al. 2025).

### Thinking through regulatory actions

Any regulatory response to concentrated 7-OH and related mitragynine-derived products should distinguish clearly between botanical kratom and high-potency, non-botanical opioids derived from the kratom scaffold. Treating these categories as equivalent—scientifically, legally, or from a public-health standpoint—obscures material differences in composition, pharmacology, and risk, and invites both under- and over-regulation.

Scheduling policy epitomizes the necessary balance. A Schedule I listing would simplify enforcement but would also impede mechanistic, clinical, and epidemiologic research at the moment such evidence is most needed. By contrast, a narrowly drawn Schedule II placement for 7-OH focused on the isolated substance and chemically enriched or semi-synthetic preparations would acknowledge its μ-opioid agonism and abuse liability. At the same time, it will preserve a viable research pathway under established registration, security, and quota systems. The listing language should explicitly exclude naturally occurring concentrations within unextracted plant material to avoid unintended restrictions on studies of botanical kratom. To mitigate research frictions further, federal guidance could provide expedited pathways for academic and public-health laboratories (streamlined DEA registration, predictable quotas for reference standards, and clear import-permit procedures).

Supply-side controls must be paired with demand-side safeguards. Abrupt removal of concentrated 7-OH from retail channels risks precipitating withdrawal among current users and driving a rapid shift to illicit markets with poorer quality control. Transitional measures like time-limited grace periods linked to point-of-sale education, naloxone co-dispensing, and facilitated referral to medications for opioid use disorder (e.g., buprenorphine) would reduce foreseeable harms while new rules take effect. In parallel, low-threshold treatment access and targeted public-health messaging should be expanded to accommodate increased help-seeking.

Marketplace standards should be differentiated by product class. For botanical kratom, a consumer-protection framework is appropriate: uniform age restrictions; plain packaging and limits on confectionary flavors/child-appealing imagery; accurate nomenclature that prohibits labeling semi-synthetic 7-OH as ‘kratom’; contaminant and identity testing with batch-level disclosure; and prominent warnings regarding dependence and drug–drug interaction risks. Concentrated 7-OH and chemically modified analogues warrant opioid-class controls irrespective of botanical origin. These could include potency caps and unit-dose limits; restrictions on dosage forms intended to bypass first-pass metabolism (e.g., intranasal or sublingual preparations) unless dispensed under medical supervision; pharmacy-only or prescriber-only distribution where any lawful access is contemplated; verified identity/purity with chain-of-custody documentation; and mandatory adverse-event reporting. Online commerce should incorporate robust age and identity verification, shipment to verified adult recipients, and platform accountability for repeated violations.

Preemptive action is justified for next-generation mitragynine derivatives (e.g., MGM-15 and structurally related dual μ/δ agonists) that were rationally engineered to enhance opioid potency and that are already appearing in ‘research-only’ yet *de facto* consumer channels. An emergency, time-limited control (with clear structural definitions) can prevent rapid diffusion while permanent rulemaking evaluates pharmacology, abuse potential, and legitimate research uses. Any permanent control should again be accompanied by research-facilitation provisions to ensure that scientific and public-health laboratories can characterize these compounds and monitor displacement effects.

Effective regulation also depends on measurement. Federal and state guidance should recommend routine inclusion of 7-OH and mitragynine-pseudoindoxyl in clinical and forensic toxicology panels, standardized coding within poison-center surveillance, and linkage of specimen results to verified product composition when feasible. These steps will improve case attribution, enable evaluation of regulatory impact, and detect substitution toward newly emergent analogues.

Finally, existing authorities outside scheduling can correct the most problematic retail practices. Coordinated FDA–FTC enforcement against unsubstantiated therapeutic claims, misbranding (including use of the ‘kratom’ label for semi-synthetic 7-OH), and child-appealing packaging would reduce exposure risk even before scheduling changes are finalized. Taken together, a targeted scheduling approach, transitional public-health supports, differentiated marketplace standards, preemptive control of high-potency analogues, strengthened analytics, and assertive marketing enforcement constitute a coherent framework that addresses near-term risk while preserving the capacity to generate the evidence needed for durable policy.

## Conclusions

The evidence reviewed here demonstrates that 7-OH products currently available in U.S. commerce are not equivalent to botanical kratom preparations. In the native plant, 7-OH is absent from fresh leaves and detectable only at trace levels in dried material or decoctions, typically <2% of the alkaloid fraction and often below analytical detection limits. Circulating 7-OH in traditional users derives almost entirely from metabolic conversion of mitragynine, constrained by formation-rate limitation. By contrast, commercial 7-OH products are chemically enriched or semi-synthetic, generated by oxidation of mitragynine isolates, and deliver amounts that far exceed natural abundance. Such preparations bear little resemblance to traditional kratom leaf or tea, despite frequent marketing under the ‘kratom’ label. Their botanical provenance therefore does not confer equivalence or continuity with kratom’s ethnobotanical history.

Against this backdrop, the safety profile of concentrated 7-OH becomes clear. Pharmacologically, 7-OH is a potent and selective μ-opioid receptor agonist with nanomolar affinity and full agonist efficacy, surpassing mitragynine and often morphine in functional potency. Preclinical models consistently demonstrate respiratory depression, tolerance, dependence, and withdrawal, aligning its toxicology with classical opioids rather than with botanical kratom. Epidemiologic signals reinforce these findings: poison center data, state health department alerts, and federal surveillance all indicate increasing exposure and morbidity linked to concentrated 7-OH products. Clinical case reports describe dependency-forming use, withdrawal syndromes, psychiatric destabilization, and, in some cases, severe self-harm. Forensic investigations confirm fatalities with postmortem 7-OH concentrations consistent with opioid overdose.

Altogether, these findings demonstrate that 7-OH products as they are currently formulated and marketed present a significant and unreasonable risk of illness and death. There is no peer-reviewed evidence to suggest that such products are safe for consumer use, no basis in the literature to support ‘generally recognized as safe’ (GRAS) status, and no precedent of traditional use at pharmacologically relevant concentrations.

In summary, concentrated 7-OH should be understood as a semi-synthetic opioid derivative, not a botanical preparation, and its risk profile is defined by predictable opioid-class toxicities. Under the common conditions of use implied by labeling and availability—oral, sublingual, or intranasal ingestion at milligram-scale doses—7-OH poses unacceptable hazards of dependence, withdrawal, respiratory depression, and death. Regulatory, public health, and forensic data converge on the conclusion that 7-OH cannot be regarded as safe for consumers and should not be conflated with kratom’s traditional use. Accordingly, regulatory responses should be targeted: a narrowly drawn control of 7-OH and related semi-synthetic analogues (e.g., Schedule II–style placement with explicit research facilitation) coupled with consumer-protection standards for botanical kratom (age restrictions, plain packaging, accurate nomenclature, contaminant testing). To reduce foreseeable harm during implementation, transitional measures such as naloxone co-dispensing, linkage to medications for opioid use disorder, and strengthened analytical surveillance that routinely includes 7-OH and mitragynine-pseudoindoxyl should accompany marketplace restrictions and enforcement against misbranding and youth-appealing marketing.

## Data Availability

This study does not have associated data.
